# Editorial: Advanced AI methods for plant disease and pest recognition

**DOI:** 10.3389/fpls.2024.1434320

**Published:** 2024-05-30

**Authors:** Jucheng Yang, Tonghai Liu, Sook Yoon, Alvaro Fuentes, Yalin Wu

**Affiliations:** ^1^ College of Artificial Intelligence, Tianjin University of Science and Technology, Tianjin, China; ^2^ College of Computer and Information Engineering, Tianjin Agricultural University, Tianjin, China; ^3^ Department of Computer Engineering, Mokpo National University, Mokpo, Republic of Korea; ^4^ Department of Electronics Engineering, Jeonbuk National University, Jeonju, Republic of Korea; ^5^ Core Research Institute of Intelligent Robots, Jeonbuk National University, Jeonju, Republic of Korea; ^6^ Lushan Botanical Garden, Chinese Academy of Sciences, Jiujiang, China

**Keywords:** plant disease, pest recognition, artificial intelligence, deep learning, disease identification, disease detection, disease segmentation

Plant diseases and pests cause significant losses to farmers and threaten food security worldwide. Monitoring the growing conditions of crops and detecting plant diseases is critical for sustainable agriculture. Traditionally, crop inspection has been carried out by people with expert knowledge in the field. However, regarding any activity carried out by humans, this activity is prone to errors, leading to possible incorrect decisions. Innovation is, therefore, an essential fact of modern agriculture. In this context, deep learning has played a key role in solving complicated applications with increasing accuracy over time, and recent interest in this type of technology has prompted its potential application to address complex problems in agriculture, such as plant disease and pest recognition. Although substantial progress has been made in the area, several challenges remain, especially those that limit systems to operate in real-world scenarios. This Research Topic aims to explore recent advanced AI methods for plant disease and pest recognition for real-world applications.

In this Research Topic, a total of 21 papers have been published, encompassing the contributions of 84 distinct authors. The content primarily delves into the realms of disease detection and identification pertaining to crops such as tomatoes, peppers, rice, corn, soybeans, alongside fruits including apples, grapes, and blueberries. Furthermore, the scope encompasses the automation of harvesting processes and anomaly detection within economically significant crops like tea and tobacco. Notably, there are also inquiries into the behavioral patterns of Diptera Tephritidae.

The focus of these studies centers on the identification, detection, and segmentation of plant diseases, as well as aspects related to harvesting, pests, and growth monitoring. Among the research papers, 18 are dedicated to the study of plant diseases, while the remaining cover various other topics. The primary subjects of investigation include leaves, fruits, and flowers of plants, with additional examinations into pests and fungi. Specifically, there are 17 papers primarily focused on leaf analysis, 3 on fruit analysis, and 1 each on pests, flowers, and fungi. From this, it is evident that the prevailing research methodologies predominantly focus on the recognition of plant diseases through leaf analysis. From a task-oriented perspective, the requirements for identification tend to be idealized, often limited to utilizing images containing a single leaf. However, as research and applications progress, achieving segmentation, detection, and optimized deployment in real-world scenarios becomes increasingly crucial. Consequently, there is a burgeoning interest in exploring the vast research potential surrounding fruits, flowers, pests, and fungi.

In identification tasks, the primary challenge lies in the feature extraction capacity of models, mainly due to the visual resemblance of different diseases across various crops. This challenge is exacerbated by the incompleteness of datasets and resource constraints during deployment. The ResNet50-DPA model (Liang and Jiang) addresses this challenge by enhancing the network structure with attention mechanisms, thereby augmenting the backbone network’s capability in extracting image features. On the other hand, attention is directed towards improving the network’s classification head in the AD approach. He et al. achieved this by training multiple models tailored to different disease recognition tasks and refining the classification results through the optimization of ensemble weights. Plant disease datasets often suffer from sample scarcity, domain shifts, and the presence of unknown classes. Lin et al. addressed the issues of sample scarcity and domain shifts through few-shot learning, while Wang tackled the problem of unknown classes using zero-shot learning. Zeng et al., on the other hand, proposed a multimodal approach. They leveraged image features to generate textual descriptions of diseases, thereby facilitating model training. This not only enhanced the quality of extracted features but also alleviated the scarcity of samples in the dataset. In-depth studies, such as Qu et al., have delved into establishing severity grading and stage progression of diseases. This approach provides valuable references for the practical deployment of fine models in specialized fields. Ning et al. reviewed the performance of lightweight models in rice disease recognition. Furthermore, Ang et al. employed structural reparameterization techniques to reduce model parameter size, facilitating practical deployment.

In segmentation tasks, the primary challenges stem from the difficulty in distinguishing between background and foreground and the presence of small yet densely packed objects. Most of these studies rely on meticulously collected and annotated datasets, which are essential prerequisites for addressing the challenges. However, various methods have been proposed to tackle these challenges. To segment tea leaf buds, Zhang et al. enhances model attention and further computes picking points for the buds during post-processing. Additionally, Ma et al. employs a series of preprocessing techniques such as Gaussian filtering to refine the image background, thereby reducing the noise that the model needs to handle. Leveraging a blend of CNN and Transformer models, Lu et al. achieved fine-grained semantic segmentation by harnessing the strengths of each approach. Building upon the UNet model, Wang et al. utilized multi-scale features to achieve granular segmentation.

In detection tasks, the primary challenges continue to arise from dataset limitations. This is because the task closely simulates real farm environments, leading to issues such as unknown classes, annotation errors, and labeling inaccuracies within the dataset. Additionally, the challenge of detecting small yet densely packed objects persist. Dong et al. proposed two approaches to address these challenges. Firstly, they introduce a method that utilizes teacher-student networks for self-supervised repair of imprecise and incomplete annotations. Secondly, they present an Open-World detection method (Dong, et al.) that combines incremental learning with open-set detection. These approaches address the major drawbacks of imperfect datasets and provide valuable insights for deployments closer to real-world applications. Similarly to segmentation tasks, methods involving attention mechanisms (Liu et al.; Lv and Su) are employed to ensure the model’s capability in detecting small and densely packed targets. Moreover, perspective correction of detection models (Pan et al.) based on drone-captured imagery offers a distinct research perspective for practical deployment.

Some innovative research endeavors also demonstrate immense potential. Reis Pereira et al., for instance, cultivated diseased plants in a controlled laboratory environment and utilizes hyperspectral imaging to predict asymptomatic diseases in advance based on disease stage. Furthermore, Wu et al. conducted semantic segmentation of fungal pathogens causing diseases. While the aforementioned detections cannot be accomplished using conventional cameras, their profound exploration of diseases provides crucial reference points for subsequent research. Insect pest detection represents another vital research direction. Zhou et al. shifted its focus towards insects, analyzing pest generation through detection, segmentation, and tracking of insect behavior.

In conclusion, regardless of identification, segmentation, or detection tasks, dataset limitations remain the primary bottleneck in the development of advanced AI methods for plant disease and pest recognition. As articulated in the perspective article (Xu et al.), imperfect datasets always entail additional risks and challenges. However, effectively leveraging these datasets can still reduce costs and enhance efficiency. For Research Topics in plant disease and pest recognition that rely heavily on domain expertise, further refining the objectives of recognition and expanding the applicability of models to minimize data dependency in real-world environments are two key objectives for future development.

## Author contributions

JY: Writing – review & editing, Writing – original draft, Supervision, Conceptualization. TL: Writing – original draft, Formal Analysis, Data curation. SY: Writing – original draft, Validation, Investigation, Conceptualization. AF: Writing – original draft, Methodology, Investigation. YW: Writing – original draft, Validation, Data curation.

